# The Association Between Family Health and Proactive Health Risk Management With the Mediating Role of Health Literacy: Nationwide Cross-Sectional Study

**DOI:** 10.2196/73659

**Published:** 2026-04-23

**Authors:** Shangfeng Tang, Yilin Wei, Heng Dong, Kangkang Zhang, Chunying Wang, Jia Song, Hua Qing, Yibo Wu

**Affiliations:** 1 School of Medicine and Health Management, Tongji Medical College, Huazhong University of Science and Technology, Hangkong Road No.13, Wuhan, Hubei, China, 86 13937920919; 2 School of Public Health, Peking University, Beijing, Beijing, China

**Keywords:** family health, health literacy, proactive health risk management, middle-aged and older adults, health behavior

## Abstract

**Background:**

Modifiable unhealthy behaviors account for over two-thirds of new cases of noncommunicable diseases. Behavioral risk factor reduction is a potentially cost-effective means to improve long-term health outcomes. Although family serves as a pivotal cornerstone for fostering and maintaining individuals’ health, the associations between family health (FH) and the proactive health risk management index (PHRMI) remain unclear.

**Objective:**

This study aimed to construct a comprehensive index to measure the PHRMI and examine the mediating effect of health literacy on the association between FH and the PHRMI, as well as the moderating effect of family communication on the associations among the PHRMI, health literacy, and FH.

**Methods:**

A cross-sectional questionnaire survey was conducted with 30,044 participants from 34 provinces or regions in China who were recruited using a multistage stratified sampling strategy from June 20, 2023, to August 31, 2023. This study constructed the PHRMI for the general population by encompassing BMI, physical activity (International Physical Activity Questionnaire–Short Form), depression (Patient Health Questionnaire-9), sleep quality (Brief version of the Pittsburgh Sleep Quality Index), smoking behavior, and drinking behavior. Further, we assessed FH (Short Form of the Family Health Scale), health literacy (Short-Form Health Literacy Questionnaire-4), and family communication (Family Communication Scale–Short Form). In addition, we collected the sociodemographic characteristics of the participants. We used model 4 of the IBM SPSS macro PROCESS to verify the mediating effect of health literacy between FH and the PHRMI, while model 7 was adopted to test the moderated mediation of family communication among the PHRMI, health literacy, and FH.

**Results:**

Higher levels of FH were significantly associated with higher PHRMI levels (β=.710, 95% CI 0.669-0.752). Health literacy significantly mediated the association between FH and the PHRMI (β=.207, 95% CI 0.168-0.245), playing a partial mediating role. Family communication significantly moderated the association between FH and health literacy (β=.117, 95% CI 0.105-0.130). The simple slope analysis showed that higher levels of family communication exacerbated the effects of FH on health literacy. Subsequently, we performed a sensitivity analysis, and the main results aligned with the findings of prior studies. Nevertheless, the subgroup analysis revealed that the mediating effect of health literacy was not significant in the group aged >60 years (β=.066, 95% CI −0.024 to 0.157).

**Conclusions:**

FH can be an important target that appears to be positively linked to proactive health risk management and health literacy. FH promotion for older adults should pay more attention to family or intergenerational communication.

## Introduction

The presence of multiple health risk factors (environmental and occupational hazards, behavioral risks, and metabolic risks) exerts a cumulative negative influence on health [1]. From the perspective of the full life cycle, in addition to genetic factors, the accumulation of risk factors during the early stages of life exacerbates health hazards in the later stages of life [2,3]. Modifiable unhealthy behaviors, such as tobacco use, consumption of foods high in saturated and trans fats, salt, and sugar, physical inactivity, and the harmful consumption of alcohol, account for more than two-thirds of all new cases of noncommunicable diseases and increase the risk of complications in individuals with noncommunicable diseases [4]. The rapid expansion and high incidence of chronic diseases worldwide, combined with population aging, have resulted in an increasing disease burden on individuals [5], families, and society [6]. Behavioral risk factor reduction is a potentially cost-effective means to improve long-term health outcomes and reduce the disease burden. Therefore, numerous countries have implemented policies to encourage individuals to actively practice a healthy lifestyle, aligning with the “proactive health” initiative actively promoted by China in recent years.

Despite being a novel concept proposed in 2015, proactive health has a lengthy research history concerning its concept and practice [7-11]. Most relevant research focuses on the connotation, subject matter, objectives, essential components, and implementation strategies of proactive health. Current academic discourse presents two main perspectives on the concept of proactive health [12]. First, based on the micro perspective of residents, proactive health is defined as guiding residents to identify and control risk factors [8], advocating self-active health management, and promoting the formation of healthy living habits [13]. Second, proactive health inherently leverages information technology to achieve its “proactive and full-lifecycle” characteristics in health management services [14]. From this perspective, proactive health is defined as health care professionals regularly monitoring residents’ health risk indicators through means such as model design and platform construction [15], aiming to detect service needs early and intervene in the medical and health service process in advance. Despite the variations in conceptualizations of proactive health, scholars in China have reached a consensus on the identification and control of health risk factors within the core essence of proactive health. Some scholars have focused on special groups, such as adolescents [16], disabled older adults [17], patients with hypertension, patients with coronary heart disease [18], and others, to develop appropriate proactive health behavior measurement scales. The development approach mainly revolves around the specific health needs or unique health risk factors of the participants being assessed. For instance, proactive health management for adolescents [16] is composed of 5 dimensions: health responsibility, physical activity, nutritional diet, mental health, and self-discipline. In line with the above studies, we developed the proactive health risk management index (PHRMI), integrating the most common health risks for the general population. Proactive health risk management is defined as a resident’s active engagement in health management activities encompassing psychological, dietary, exercise, sleep, and lifestyle aspects. It involves identifying and modifying detrimental habits to prevent health risks from the source, encouraging individuals to assume health management responsibilities, thereby comprehensively safeguarding individual and collective well-being. Risk factors were extracted based on the “four cornerstones of health” outlined in the “Victoria Declaration,” namely “balanced diet,” “moderate exercise,” “smoking cessation and alcohol restriction,” and “psychological balance” [19]. In addition, sleep of sufficient quality and duration is essential for good health [20] and reduces the risk of total mortality, obesity [21], cardiovascular disease [22,23], cerebrovascular diseases [24,25], and dementia. Therefore, sleep quality is incorporated as one of the components of the proactive health index.

Subsequently, this study aims to investigate family-level and individual-level determinants influencing the PHRMI. Entering the era of “Public Health 3.0,” the family’s role as a fundamental contributor to individual and community health remains unquestioned. The family, owing to its fundamental capacity to nurture, care for, protect, educate, and influence across the lifespan, serves as a pivotal cornerstone for fostering and maintaining individuals’ health. Engaging families as proactive participants in health promotion initiatives can enhance family members’ acceptance and use [19] and may facilitate longer-lasting and larger-scale behavioral changes [26]. Ho et al [27] have developed a more comprehensive conceptual framework for “family health” (FH) by using the Delphi method. The framework posits FH as a multifaceted resource arising at the family-unit level through the interplay of the members’ health, their interactions, and the family’s array of physical, social, emotional, economic, and medical resources. FH is a comprehensive construct that encompasses intrafamily dynamics, such as associations, communication, and the domestic environment, as well as the accessibility of social support and health resources. The influence of FH on proactive health, both in terms of its presence and magnitude, remains uncertain. Clarifying these ambiguities may have important implications for individual, clinical, and public health, especially amid the escalating incidence of chronic diseases [28].

Health literacy serves as a key measure reflecting an individual’s accumulation of health knowledge and capabilities [29]. It is hypothesized that FH would result in a positive influence on health literacy, given that the family constitutes a primary environment through which health-related attitudes, behaviors, and competencies are developed. Existing studies have demonstrated that individuals with low health literacy are associated with inadequate health-related behaviors [30], leading to adverse health-related outcomes [31], including poorer general health [32], more hospitalizations [33], compromised self-management ability, less effective use of health services [34], and higher health care costs [35]. Although lower health literacy is often linked to poorer health outcomes, some patients with chronic diseases can successfully manage their conditions despite inadequate health literacy. The buffering effect of valuable resources and support from social networks appears to mitigate the negative impacts of low health literacy [36]. Humans, inherently endowed with social attributes, consistently inhabit a collective milieu characterized by diverse levels of social support and resource availability. Family-level resources and support are pivotal in an individual’s social network. Research across Brazil, the United States, and China has established that family structure, income levels, and cohesion, along with other factors, significantly influence the development and maintenance of health literacy [37-39].

The enhancement of health literacy through FH promotion does not occur spontaneously but rather through dynamic interactions within familial systems. The circumplex model emphasizes that family communication is considered a facilitating dimension and is essential for family cohesion and flexibility [40]. Conversation-oriented communication patterns significantly facilitate intermember health information exchange [41]. According to the “stimulus-awareness-internalization” pathway, family communication serves as a “stimulus” by facilitating information exchange and sharing [41-43]. This process enables individuals to receive health information and resources transmitted at the family level, subsequently internalizing them into cognitive components such as health literacy. Research has shown that better family communication is associated with better health status and higher cervical cancer literacy scores [37]. Another anorexia family therapy trial showed that family communication shifting from covert aggression to overt aggression during systemic family therapy significantly improved patients’ eating attitudes [44]. The positive transformation of patients’ eating attitudes and self-health cognition was promoted by coordinating the conflicts of health concepts within the family. Therefore, this study attempts to examine whether family communication plays a moderating role in the association between FH and health literacy.

Well-established evidence indicates that over two-thirds of the risk factors for chronic diseases stem from modifiable unhealthy behaviors [2,4,45]. Therefore, encouraging individuals to adopt a healthy lifestyle is crucial and urgent, with FH potentially serving as an effective intervention target. This study explores whether and how FH can enhance the PHRMI in China. We propose the following three hypotheses: (1) FH is positively associated with proactive health risk management, (2) the association between FH and the PHRMI is mediated by health literacy, and (3) family communication moderates the mediating effect of health literacy between FH and the PHRMI.

## Methods

### Sampling and Participants

The data originated from a nationwide, representative, population-based survey conducted from June 20, 2023, to August 31, 2023, encompassing 150 cities from 23 provinces, 5 autonomous regions, 4 municipalities directly under the Central Government, and 2 special administrative regions in China [46,47]. To ensure the overall representativeness of the study population, the survey used a multistage sampling method, incorporating stratified sampling at various levels, including province, city, and community. During the first stage, the city selection included capital cities across China, and 2 to 12 cities were selected from each noncapital prefecture within every province and autonomous region, totaling 150 cities. The number of cities selected from each province was determined by the population size weight of that province. Moving on to the second stage, of the 150 municipalities sampled, the number of communities sampled within each municipality was proportional to the population base of the first-level administrative district. A total of 10 to 60 communities were sampled, with an urban-to-rural ratio of 3:2 in the municipalities sampled in each province, resulting in 800 communities. In the last stage, quota sampling was used at the community and village levels as well as at the individual level, using quotas that were determined based on sex and age attributes from the data of “The 7th National Census in 2021.” The sample size was estimated to be 45,830. After implementing quota sampling, the final sample included 30,054 participants. This sample specifically accounted for attributes such as sex and age.

The survey protocol has been published [48]. Inclusion criteria for our survey include: (1) being aged 18 years or older, (2) holding the nationality of the People’s Republic of China, (3) being permanent residents with annual travel of 1 month or less, (4) being able to complete the online questionnaire independently or with assistance, and (5) being able to understand each questionnaire item. Individuals who were delirious, suffering from cognitive dysfunction, participating in other similar research, or unwilling to participate were excluded. In this study, we excluded samples with missing data on critical study variables. Specifically, 10 participants were removed due to incomplete bedtime and wake-up time records, which precluded reliable assessment of their sleep quality.

### Ethical Considerations

This study was conducted in compliance with the ethical guidelines of the Declaration of Helsinki and received approval from the ethics research committee of the Shandong Provincial Hospital (SWYX:NO 2023‐198). All data in this research did not involve participant compensation. All participants were required to sign informed consent prior to participating in the study. Meanwhile, we ensured the confidentiality of the collected data through anonymity and stringent security measures.

### Proactive Health Risk Management Index

**Figure 1.**
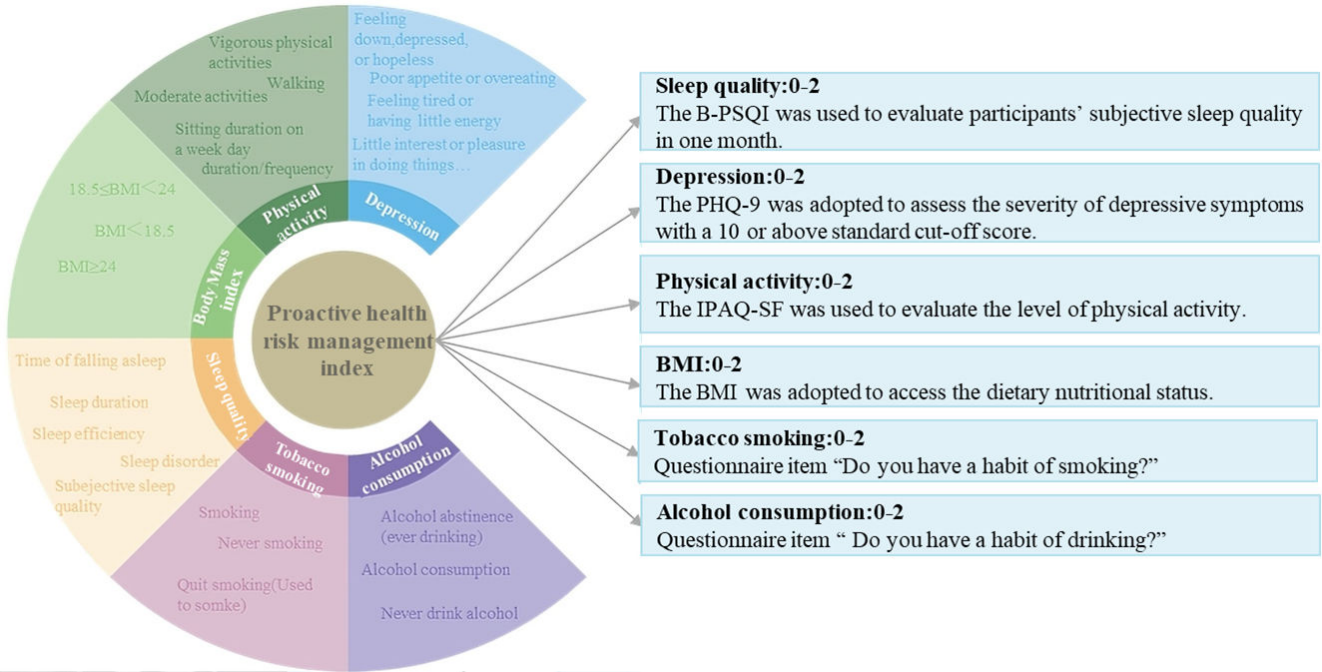
Schematic diagram of the components of proactive health risk management, encompassing sleep quality (Brief version of the Pittsburgh Sleep Quality Index [B-PSQI]), depression (Patient Health Questionnaire-9 [PHQ-9]), physical activity (International Physical Activity Questionnaire–Short Form [IPAQ-SF]), BMI, tobacco smoking, and alcohol consumption.

Referring to previous relevant studies [49-51], PHRMIs were measured through a self-created comprehensive instrument, including BMI, physical activity, depression, sleep quality, smoking behavior, and drinking behavior. We defined the PHRMI as comprising 6 aspects, with its measurement based on the 4 cornerstones of health proposed by the World Health Organization. Given the detrimental health effects of tobacco smoking and alcohol consumption, we incorporated questions about smoking and drinking behavior, offering response options such as 0 (“yes, smoking or drinking”), 1 (“ever smoked or drank [quit]”), and 2 (“never smoked or drank”), alongside “Do you have a habit of smoking?” and “Do you have a habit of drinking?” The International Physical Activity Questionnaire–Short Form (IPAQ-SF) was used to evaluate the level of physical activity, assessing participants’ vigorous activity, moderate activity, and walking activities in terms of frequency and duration from the previous week [52,53]. Previous studies have indicated a moderate-to-high reliability of IPAQ-SF across 12 countries, as well as the Chinese version. According to each participant’s total metabolic equivalent–minute/week in walking, moderate, and vigorous activity for 7 days, we categorized them as high, moderate, and low physical activity and assigned the values “2,” “1,” or “0,” respectively. The 9-item Patient Health Questionnaire was adopted to assess the severity of depressive symptoms [54], using a cutoff score of 10 or higher. We encoded Patient Health Questionnaire-9 scale scores of “15 or more” as “0,” “5 to 14” as “1,” and “less than 4” as “2.” The Brief Version of the Pittsburgh Sleep Quality Index (B-PSQI) was used to evaluate participants’ subjective sleep quality over 1 month [55], and it has been proven to be a reliable and valid sleep-quality measure for the general population. This scale encompassed sleep efficiency, sleep latency, sleep duration, sleep disorders, and sleep quality, with a 5 or higher standard cutoff score. We encoded B-PSQI scale scores of “5 or more” as 0 and “less than 5” as 2. BMI was selected as an indicator for the examination of reasonable diet. Participants were classified as overweight (BMI≥24 kg/m^2^), normal weight (18.5 kg/m^2^≤BMI<24 kg/m^2^), and underweight (BMI<18.5 kg/m^2^), respectively. The indicator was coded as 0 (overweight or underweight) or 2 (normal weight). As shown in Figure 1, the total scores were summed to calculate a PHRMI score ranging from 0 to 12, where a higher score signifies better proactive health risk management.

### Assessment of FH

FH was measured using the Short Form of the Family Health Scale [56], which was modified to align with China’s national conditions and cultural characteristics. The scale has been validated and proven to be reliable and effective. It contains four dimensions: (1) family healthy lifestyle; (2) FH resources; (3) family, social, or emotional health processes; and (4) family external social support. The 3 dimensions (7 items) are scored on a Likert-5 scale ranging from 1 (strongly disagree) to 5 (strongly agree), except for “family health resources” (3 items). Scores ranged from 10 to 50 points, with a high score indicating higher levels of FH. The Cronbach α for the sample was 0.807, and the Kaiser-Meyer-Olkin value was 0.879, both indicating acceptable reliability and validity.

### Assessment of Health Literacy

The mediator variable in this study was health literacy, assessed by a 4-item version of the Short-Form Health Literacy Questionnaire-4. The simplified health literacy scales, derived from the Short-Form Health Literacy Questionnaire-12 [57], demonstrated good reliability and validity and were reliable and effective tools for assessing the health literacy of the Chinese population. The total score ranges from 4 to 16, with a higher score indicating better levels of health literacy.

### Assessment of Family Communication

Family communication was the moderator in this study, assessed using the Family Communication Scale-Short Form (FCS-SF). For example, participants were asked, “Family members can discuss things calmly with each other.” Items were scored on a 5-point scale from 1 to 5, from 1 (“strongly disagree”) to 5 (“strongly agree”). The sum of scores on the FCS-SF, ranging from 4 to 20 points, indicates better communication between family members with higher scores. The Cronbach α for the FCS-SF was 0.926 in this study.

### Covariates

Covariates were identified initially based on relevant research and general knowledge, including age, sex, nationality, education level, family type, and employment status. Enrolled covariates were analyzed subsequently for intergroup differences using the bilateral 2-tailed Student *t* test or one-way ANOVA. Covariates were considered for inclusion as potential confounders in the final models if significant differences between the groups were observed. The final demographic characteristics included age, sex (1=“male” and 2=“female”), nationality (1=“Han nationality” and 2=“nationality minority”), educational level (1=“illiterate or semi-illiterate”; 2=“junior high school and below”; 3=“secondary technical school, senior high school, and college for professional training”; and 4=“Bachelor’s degree and higher”), residence (1=“urban” and 2=“rural”), family type (1=“core family,” 2=“backbone family,” 3=“joint family,” 4=“conjugal family,” 5=“single-parent family,” and 6=“other family”), household income monthly per capita (1=“≤￥3000” [US $421], 2=“￥3001-￥6000” [US $421-US $841], and 3=“≥￥6001” [US $841]), children (0=“no” and 1=“yes”), and insurance (0=“no” and 1=“yes”). Age and self-assessment of social status (scoring 0-7) were set as continuous variables.

### Statistical Analysis

Demographic characteristics, FH, PHRMIs, health literacy, and family communication of participants were summarized using frequencies (percentages) or means and SDs. The disparity in PHRMIs across different groups was analyzed using the 2-tailed Student *t* test and one-way ANOVA. We used SPSS 26.0 (IBM Corp) and the Hayes [58] SPSS macro program PROCESS to test the mediating effect using model 4. If the coefficient *a* (representing the association between X and M), *b* (the mediator’s effect on Y), and *c* (the relationship between X and Y) in the regression model including X, M, and Y were statistically significant, this indicated that the mediating effect was significant. Next, we constructed a new model containing the aforementioned variables and the product term of the moderator and independent variable to verify the moderating effect of family communication. Finally, the PROCESS macro for SPSS was used to examine whether family communication moderated the direct and indirect effects of FH on the PHRMI. All statistical analyses were conducted using SPSS 26.0. In addition, all models controlled for covariates, and the significance level was set at a *P* value of <.05 for all the hypothesis tests [59].

### Sensitivity and Subgroup Analysis

To validate the results, we conducted a sensitivity analysis. Specifically, we replaced depression with generalized anxiety disorder as a measure of the mood component and recalculated the PHRMI. Following this adjustment, we repeated the aforementioned statistical analysis steps to ascertain whether the direct and indirect effects remained statistically significant. In addition, subgroup analysis was used to explore the heterogeneity of PHRMIs. Considering the wide age spectrum of participants (aged 18-106 y), we further stratified the age cohorts to compare the association between familial health and proactive health levels across different age groups, as well as to examine the mediating role of health literacy.

## Results

### Sample Characteristics

**Figure 2.**
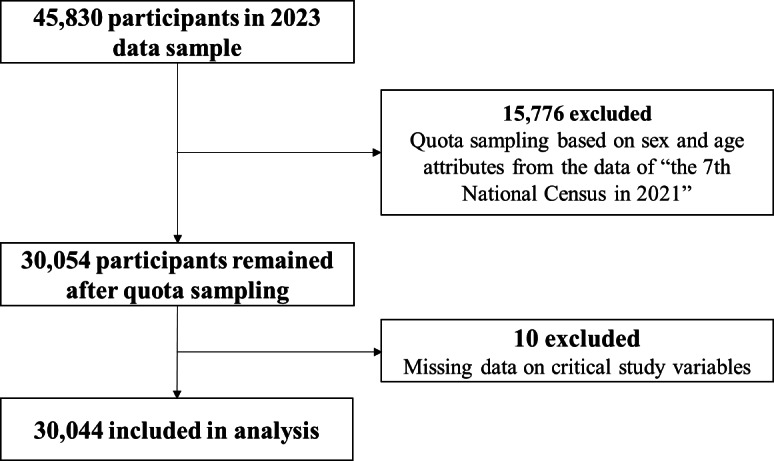
Flowchart of participant selection in the 2023 Psychology and Behavior Investigation of Chinese Residents for this study, conducted from June 20, 2023, to August 31, 2023.

The process for screening participants is shown in Figure 2. A total of 30,044 participants were included in the final analysis. All participants had a mean age of 43.99 (SD 16.55) years, ranging from 18 to 106 years, with 15,004 (49.90%) males and 15,040 (50.10%) females. Most participants were married (n=19,391, 64.50%), lived in urban areas (n=20,728, 69.00%), and reported having a Bachelor’s degree or higher (n=10,609, 35.30%). The mean scores for FH and the PHRMI were 39.86 (SD 6.15) and 5.88 (SD 1.78), respectively. Subsequently, we performed an analysis of intergroup differences to assess the disparity in PHRMIs across demographic variables, using a 2-tailed *t* test and one-way ANOVA. Significant differences were found among various demographic groups, including sex, nationality, education level, and residence (Table 1).

**Table 1.** Characteristics of participants and the scores of family health (FH) and the proactive health risk management index (PHRMI) based on demographic variables in this cross-sectional study (2023, China; N=30,044).

Characteristics	Total, n (%)	FH, mean (SD)	PHRMI, mean (SD)	*P* value
Age (y)	42.92 (16.56)	3.99 (0.61)	7.57 (2.29)	<.001
Sex				<.001
Male	15,004 (49.9)	3.95 (0.63)	7.08 (2.43)	
Female	15,040 (50.1)	4.02 (0.60)	8.05 (2.03)	
Nationality				<.001
Han	27,290 (90.8)	4.00 (0.61)	7.62 (2.26)	
National minority	2754 (9.2)	3.85 (0.65)	7.08 (2.50)	
Education level				<.001
Illiterate or semi-illiterate	1458 (4.9)	3.83 (0.61)	7.54 (2.09)	
Junior high school and below	7783 (25.9)	3.92 (0.59)	7.57 (2.25)	
Secondary technical school, senior high school, and college for professional training	10,194 (33.9)	3.98 (0.62)	7.47 (2.33)	
Bachelor degree and higher	10,609 (35.3)	4.06 (0.62)	7.66 (2.31)	
Family type				<.001
Core family	15,141 (50.5)	4.06 (0.59)	7.77 (2.27)	
Backbone family	6066 (20.2)	3.97 (0.60)	7.49 (2.22)	
Joint family	1220 (4.1)	3.92 (0.62)	7.19 (2.40)	
Conjugal family	4963 (16.5)	3.90 (0.62)	7.42 (2.33)	
Single-parent family	1047 (3.5)	3.87 (0.63)	7.04 (2.28)	
Other	1567 (5.2)	3.75 (0.68)	7.04 (2.37)	
Employment status				<.001
Incumbent	13,295 (44.3)	4.03 (0.61)	7.60 (2.35)	
Rolling stone	5042 (16.8)	3.92 (0.58)	7.44 (2.31)	
Retirement	4226 (14.1)	3.95 (0.64)	7.46 (2.18)	
Unemployment	7481 (24.9)	3.98 (0.62)	7.65 (2.24)	
Per capita monthly household income				<.001
≤￥3000 (≤US $421)	8779 (29.2)	3.84 (0.61)	7.37 (2.29)	
￥3001-￥6000 (US $421-US $841)	13,425 (44.7)	4.00 (0.60)	7.64 (2.27)	
≥￥6001(≥ US $841)	7840 (26.1)	4.13 (0.61)	7.65 (2.32)	
Children				<.001
No	10,483 (34.9)	3.96 (0.65)	7.57 (2.33)	
Yes	19,561 (65.1)	4.00 (0.60)	7.56 (2.27)	
Marital status				.105
Married	19,391 (64.5)	4.00 (0.60)	3.95 (0.64)	
Divorced, widowed, or unmarried	10,653 (35.5)	3.95 (0.64)	5.86 (1.81)	
Insurance				<.001
No	1441 (4.8)	3.74 (0.67)	5.67 (1.76)	
Yes	28,603 (95.2)	4.00 (0.61)	5.89 (1.78)	
Residence				<.001
Urban	20,728 (69)	4.02 (0.61)	7.59 (2.30)	
Rural	9316 (31)	3.91 (0.61)	7.52 (2.28)	
Self-assessment of social status	3.98 (1.36)	3.99 (0.61)	7.57 (2.29)	<.001

### Mediation Analyses

**Figure 3.**
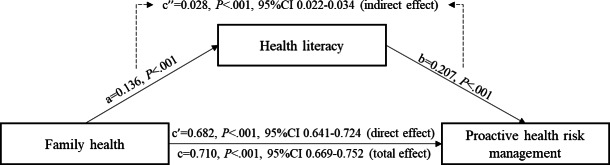
Mediating model of the effect of family health on proactive health risk management via health literacy in this cross-sectional study based on the Psychology and Behavior Investigation of Chinese Residents (2023; N=30,044). “a” represents the coefficient between family health and health literacy; “b” represents the coefficient between health literacy and proactive health risk management; and “c” represents the total coefficient when combined direct coefficient (c’) and from family health and proactive health risk management and indirect coefficient (c”) between them via health literacy.

Model 4 is a simple mediating model in SPSS macro PROCESS compiled by Hayes. We adopted to test the mediating effect of health literacy on the association between FH and the PHRMI, while controlling for age, gender, nationality, education level, family type, employment status, income, children, marital status, insurance, residence, self-assessment of social status, and registered permanent residence. Model 1 of Table 2 shows that the positive predictive effect of FH on the PHRMI was significant (β=.710, 95% CI 0.669-0.752). Model 2 of Table 2 shows that FH had a significant positive predictive effect on health literacy (β=.136, 95% CI 0.124-0.148), while health literacy also had a significant positive predictive effect on the PHRMI (β=.207, 95% CI 0.168-0.245). Moreover, when the mediating variable was added, the direct predictive effect of FH on the PHRMI was still significant (β=.682, 95% CI 0.641-0.724), as shown in model 3 in Table 2. In addition, the upper and lower bounds of the bootstrapped 95% CI for the direct effect of FH on proactive health risk management and the mediating effect of health literacy did not include zero in Figure 3, indicating that the mediating effect was significant (indirect effect=0.028*; P*<.001). The results implied that the association between FH and the PHRMI was partially mediated by health literacy.

**Table 2.** Results of the mediating effect of health literacy in the association between family health (FH) and the proactive health risk management index (PHRMI) in the cross-sectional study based on the 2023 Psychology and Behavior Investigation of Chinese Residents (N=30,044). These models incorporated covariates, including age, gender, nationality, education level, family type, employment status, income, children, marital status, insurance, residence, self-assessment of social status, and registered permanent residence.

Predictors	Model 1^a^ (DV^b^=PHRMI)	Model 2^c^ (DV=HL^d^)	Model 3^e^ (DV=PHRMI)
FH, β (95% CI)	.710 (0.669-0.752)	.136 (0.124-0.148)	.682 (0.641-0.724)
HL, β (95% CI)	—^f^	—	.207 (0.168-0.245)

a *F*_13, 30,030_=166.605, *P*<0.001, *R*2=0.109.

b DV: dependent variable.

c *F*_13, 30,030_=226.421, *P*<0.001, *R*2=0.142.

d HL: health literacy.

e *F*_14, 30,029_=164.701, *P*<0.001, *R*2=0.112.

f Not applicable.

### Moderated Mediation Analyses

**Figure 4.**
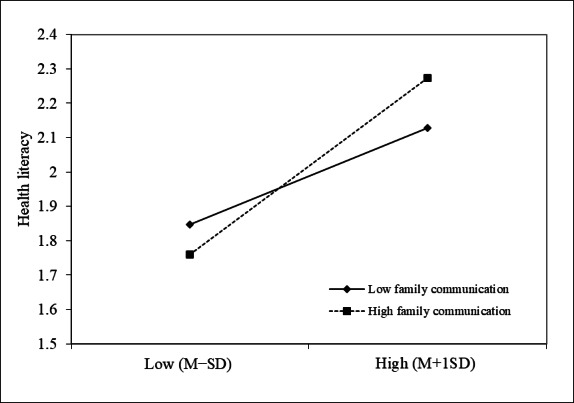
Simple slope analysis shows that family communication moderated the relationship between family health and health literacy in this cross-sectional study (2023, China; N=30,044). The function was graphed for 2 levels of the independent variable and moderator: 1 SD above the mean and 1 SD below the mean.

To verify the moderated mediation model while controlling for all covariates, we used model 7 of the SPSS macro PROCESS compiled by Hayes. The results of the family communication moderation test are shown in Table 3 and Figure 4. After putting family communication into the model, the product (interaction term) of FH and family communication had a significant predictive effect on health literacy (β=.117, 95% CI 0.105-0.130), as shown in model 4 of Table 3. The result showed that family communication played a moderating role in the association between FH and health literacy. Higher levels of family communication exacerbated the effects of FH on health literacy, as evidenced by the fact that the linear slope for higher levels of family communication is steeper than that for lower levels in Figure 4. Furthermore, the effect size estimate for the moderated mediating effect was 0.024, and the 95% CI was (0.019-0.031), excluding 0, which also supported the existence of a moderated mediation effect in this model.

**Table 3.** Results of the moderated mediation model of family communication and health literacy in the association between family health (FH) and the proactive health risk management index (PHRMI) in this cross-sectional study (2023, China; N=30,044). All models incorporated covariates.

Predictors	Model 4^a^ (DV^b^=HL^c^), β (95% CI)	Model 5^d^ (DV=PHRMI^e^), β (95% CI)
FH	.140 (0.126-0.153)	.682 (9.641-0.724)
HL	^—f^	.207 (0.168-0.245)
FC^g^	.029 (0.019-0.153)	—
FH×FC^h^	.117 (0.105-0.130)	—

a *F_15, 30,028_*=224.369; *R*2=0.152.

b DV: dependent variable.

c HL: health literacy.

d *F_14, 30,029_*=164.701; *R*2=0.112.

e PHRMI:proactive health risk management index

f Not applicable.

g FC: family communication.

h FH×FC: interaction of family health and family communication.

### Sensitivity Analysis

In the sensitivity analysis (Table S2 in Multimedia Appendix 1), we assessed proactive health risk management using generalized anxiety disorder instead of depression. The result was consistent with that of the aforementioned analysis. Upon replacing depression with generalized anxiety disorder, the results revealed that better FH was associated with better health literacy (β=.198, 95% CI 0.160-0.236) and further increased proactive health risk management (β=.630, 95% CI 0.589-0.671), with a significant mediating effect. The moderating influence of family communication on the association between FH, health literacy, and the PHRMI was not assessed in this study, as variations in proactive health risk management did not affect the moderating effect of family communication.

### Subgroup Analysis

To clarify further the association between FH and the PHRMI, we performed subgroup analyses in different age groups, including “aged 18‐44 years,” “aged 45‐59 years,” and “aged 60 years and older.” First, the positive associations between FH and the PHRMI were significant in all subgroups (for those aged 18‐44 y: β=.708, 95% CI 0.650-0.767; for those aged 45‐59 y: β=.653, 95% CI 0.574-0.731; for those aged ≥60 y: β=.732, 95% CI 0.637-0.827). Notably, the mediating effect of health literacy remained significant in individuals aged 18 to 44 and 45 to 59 years. However, for those aged 60 years or older, the association between health literacy and the PHRMI (β=.073, 95% CI −0.016 to 0.161) and the mediating effect of health literacy between FH and the PHRMI (β=.066, 95% CI −0.024 to 0.157) was not significant. The details are presented in Table S2 in Multimedia Appendix 1.

## Discussion

### Principal Findings

This study revealed that increased FH was linked to an increased PHRMI (β=.710; *P*<.001). Health literacy acted as a mediator in this relationship (β=.207; *P*<.001). Notably, while both the direct and indirect paths reached statistical significance, their magnitudes differed substantially. The strong direct association of FH with the PHRMI dominated the mediation pathway. Furthermore, higher-level family communication shows a small but statistically significant positive association with health literacy (β=.029; *P*<.001) while simultaneously intensifying the promoting effect of FH on it (β=.117; *P*<.001).

### Comparison With Prior Work

The study confirms a significant effect of family elements on an individual’s health behaviors or outcomes. This aligns with the findings of most existing studies. Previous studies have already shown that various elements of family, such as family structure [60], sibling relationships [61], family social status [62], FH climate [63], family social capital [64], and so on, influence an individual’s health. A similar effect also reflects on the influence on health behavior [65-68]. However, few studies have integrated family-level socialization dynamics, as described in family system theories [69], and explored the association between them and health or health behaviors.

This critical deviation may lead to misjudging the role of family in members’ health, limiting our ability to develop health promotion programs, policies, and goals and even blurring intervention priorities [70]. In this study, we used FH, encapsulating structure, function, and social network, which moves beyond examining isolated family factors to capture the holistic, systemic dynamics emphasized in family system theories. We discovered the positive association between FH and the PHRMI, aligning with a prior study [38]. In contrast, this study aims to examine the association between FH and clustered health behaviors, while the other focuses on exploring the mediating role of four independent health behaviors between FH and frailty. Currently, little research has examined how family-level integrated elements affect clustered health behaviors. A key innovation of this study is the construction of the PHRMI, encompassing important health risk factors. To some extent, it decreases the interdependencies among health behaviors that are often overlooked in single health behavior research. Consequently, this strengthens the argument that fostering overall FH may be a potent strategy for promoting proactive health behavior changes [71,72].

Higher health literacy is associated with increased participation in healthy behavior [30]. This research extends the outcome variables of health literacy by demonstrating that health literacy is positively associated with the PHRMI. In addition, individuals with lower FH tend to exhibit lower health literacy. Another cross-sectional survey in China also confirmed the positive association between FH and health literacy [73]. One possible explanation is that individuals with fewer family resources or family support may lack confidence and thus be unable to independently address health difficulties and manage health risk factors. In addition, health literacy mediates the positive association between FH and the PHRMI. Notably, the mediation analysis showed that the mediator only accounted for 4% of the total effect of FH on the PHRMI, suggesting a relatively modest mediated effect. FH is a complex and comprehensive concept influenced by multiple factors across family and individual domains. Complex familial interactions affect and shape individual health behavior through a variety of underlying mechanisms [74]. While our findings support the association between health literacy and proactive health risk management, exploring additional clinical, psychological, or physiological pathways remains an important direction for future research. On the other hand, family communication moderated the mediating effect of health literacy between FH and the PHRMI. This means the positive association between FH and health literacy was enhanced as family communication levels increased. The circumplex model proposes that families with a balance of cohesion and communication are more likely to set and achieve goals [75] by effectively integrating family members’ individuality and independence. Therefore, a focus on both the family and the individual for health promotion could be more effective than efforts targeted solely at the individual.

Regarding the effects of variation among different age groups, subgroup analysis results showed that FH had a stronger positive effect on the PHRMI among older adult individuals compared to younger ones. Presumably, this result is due to the fact that older adults receive health information from a relatively singular source [76,77]. As age increases, due to both subjective and objective factors such as the lack of credible internet-based health information sources [78], low-level electronic health literacy [79,80], and declines in cognitive [81,82] and physical function [83], the ability of older adults to discriminate health information gradually declines [84]. Therefore, they prefer to trust health interventions recommended by trusted family members [85]. In addition, consistent with previous studies, males tend to exhibit poorer health behaviors. Males’ PHRMI scores are lower than females’ at any age, indicating the need to strengthen males’ health interventions in particular. We find that low self-assessed social status and resource-limited economic status are associated with lower FH and PHRMIs. Self-assessed social status was statistically associated with health literacy and the PHRMI, which is consistent with previous studies**.** Our study provides further empirical evidence that health interventions are not only about health but also about improving one’s economic and social status. For instance, evidence from China shows that the national program to stabilize poverty alleviation has a significant health benefit, particularly for marginal poverty-prone households and people in resource-limited areas.

### Limitations

There are some limitations to this study that need further investigation in future research. First, although this study designed the proactive health risk management score using validated scales and indicators, the weighting coefficients between indicators were not addressed in the calculation process. Second, the cross-sectional research design means that causality cannot be deduced from the results. Future research could adopt a longitudinal design to explore the causal association between FH and proactive health risk management. Ultimately, some of the scales used to construct the proactive health risk management score in this paper, such as the B-PSQI and the IPAQ-SF, were completed by patients relying on their recall; this may introduce bias compared to the real situation. Future research could consider collecting objective indicators for the exercise and sleep dimensions.

### Conclusions

In conclusion, FH can be an important intervention target that appears to be positively linked to proactive health risk management. The mediating role of health literacy suggests its potential as a pathway through which improved FH may correspond to enhanced proactive health risk management. Our study provides evidence that maintaining FH is advantageous for promoting healthy behavior in Chinese individuals, particularly within the older adult population. Meanwhile, in the context of the family, health interventions should address a wider range of familial factors, such as family communication, family healthy lifestyle, FH resource accessibility, and family external social support, rather than focusing solely on health-related interventions.

## Supplementary material

10.2196/73659 Multimedia Appendix 1 Additional data tables.

## Abbreviations

B-PSQI: Brief version of the Pittsburgh Sleep Quality Index
FCS-SF: Family Communication Scale-Short Form
FH: family health
IPAQ-SF: International Physical Activity Questionnaire—Short Form
PHRMI: proactive health risk management index
