# Supplementary material for: The Association Between Family Health and Proactive Health Risk Management With the Mediating Role of Health Literacy: Nationwide Cross-Sectional Study
Source: JMIR Public Health Surveill. 2026 Apr 23;12:e73659. doi: 10.2196/73659 (PMC13105423; doi:10.2196/73659)
Supplement: Multimedia Appendix 1 [file publichealth-v12-e73659-s001.docx]

**Multimedia Appendix 1**

Table S1.Results of the mediating effect of health literacy in the relationship between family health and new proactive health behavior (constructed by replacing depression with anxiety)

| Predictors | Model 1^a^ (DV=PHRMInew^b^) | Model 2^c^ (DV=HL^d^) | Model 3^e^ (DV=PHBnew) |
| --- | --- | --- | --- |
| FH^f^, β (95%CI) | 0.657 (0.616,0.698) | 0.136 (0.124,0.148) | 0.630 (0.589,0.671) |
| HL, β (95%CI) | N/A^g^ | N/A^g^ | 0.198 (0.160,0.236) |

Note. N=30044.These models incorporated covariates, including age, gender, nationality, education level, family type, employment status, income, children, marital status, insurance, residence, self-assessment of social status, and registered permanent residence. ^a^F=158.589***, R^2^=0.104; ^b^PHRMInew: Proactive health risk management index, which constructed by replacing depression with anxiety; ^c^F=226.421***, R^2^=0.142; ^d^HL:Health literacy; ^e^F=156.758***, ^R2^=0.107; ^f^FH: Family health; ^g^N/A: not applicable.

Table S2. Results of the moderated mediation model of family communication and health literacy in the relationship between family health and PHRMI among different age group.

| Predictors | Model 1 (DV=PHRMI) | Model 2 (DV=HL) | Model 3 (DV=PHRMI) | Model 4 (DV=HL) |
| --- | --- | --- | --- | --- |
| **Age: 18-44 years (Adjusted by all covariates)** | | | |  |
| FH, β (95%CI) | 0.708 (0.650,0.767) | 0.208 (0.191,0.225) | 0.655 (0.596,0.714) | 0.210 (0.192,0.229) |
| HL, β (95%CI) | N/A | N/A | 0.256 (0.202,0.309) | N/A |
| FC, β (95%CI) | N/A | N/A | N/A | 0.036 (0.023,0.050) |
| FH×FC, β(95%CI) | N/A | N/A | N/A | 0.128 (0.112,0.145) |
| F | 79.590 | 59.311 | 80.378 | 65.274 |
| R^2^ | 0.097 | 0.074 | 0.102 | 0.088 |
| **Age: 45-60 years (Adjusted by all covariates)** | | | |  |
| FH, β (95%CI) | 0.653 (0.574,0.731) | 0.124 (0.101,0.147) | 0.624 (0.546,0.703) | 0.118 (0.093,0.143) |
| HL, β (95%CI) | N/A | N/A | 0.229 (0.156,0.302) | N/A |
| FC, β (95%CI) | N/A | N/A | N/A | 0.038 (0.018,0.058) |
| FH×FC, β(95%CI) | N/A | N/A | N/A | 0.105 (0.080,0.130) |
| F | 61.51 | 40.628 | 60.735 | 40.647 |
| R^2^ | 0.139 | 0.096 | 0.140 | 0.104 |
| **Age: >=60 years (Adjusted by all covariates)** | | | |  |
| FH, β (95%CI) | 0.732 (0.637,0.827) | -0.007 (-0.035,0.022) | 0.733 (0.638,0.828) | 0.012 (-0.020,0.045) |
| HL, β (95%CI) | N/A | N/A | 0.066 (-0.024,0.157) | N/A |
| FC, β (95%CI) | N/A | N/A | N/A | -0.010 (-0.038,0.017) |
| FH×FC, β(95%CI) | N/A | N/A | N/A | 0.099 (0.069,0.129) |
| F | 40.674 | 43.574 | 39.004 | 42.152 |
| R^2^ | 0.144 | 0.153 | 0.144 | 0.16 |
